# Supplementary material for: Causal Relationship between Aging and Anorexia Nervosa: A White-Matter-Microstructure-Mediated Mendelian Randomization Analysis
Source: Biomedicines. 2024 Aug 16;12(8):1874. doi: 10.3390/biomedicines12081874 (PMC11351342; doi:10.3390/biomedicines12081874)
Supplement: Supplementary file 1 [file biomedicines-12-01874-s001.zip › Supplementary Figure/Figure S4/Singe SNP plot of MR analysis for TL to ubm-b-1690.pdf]

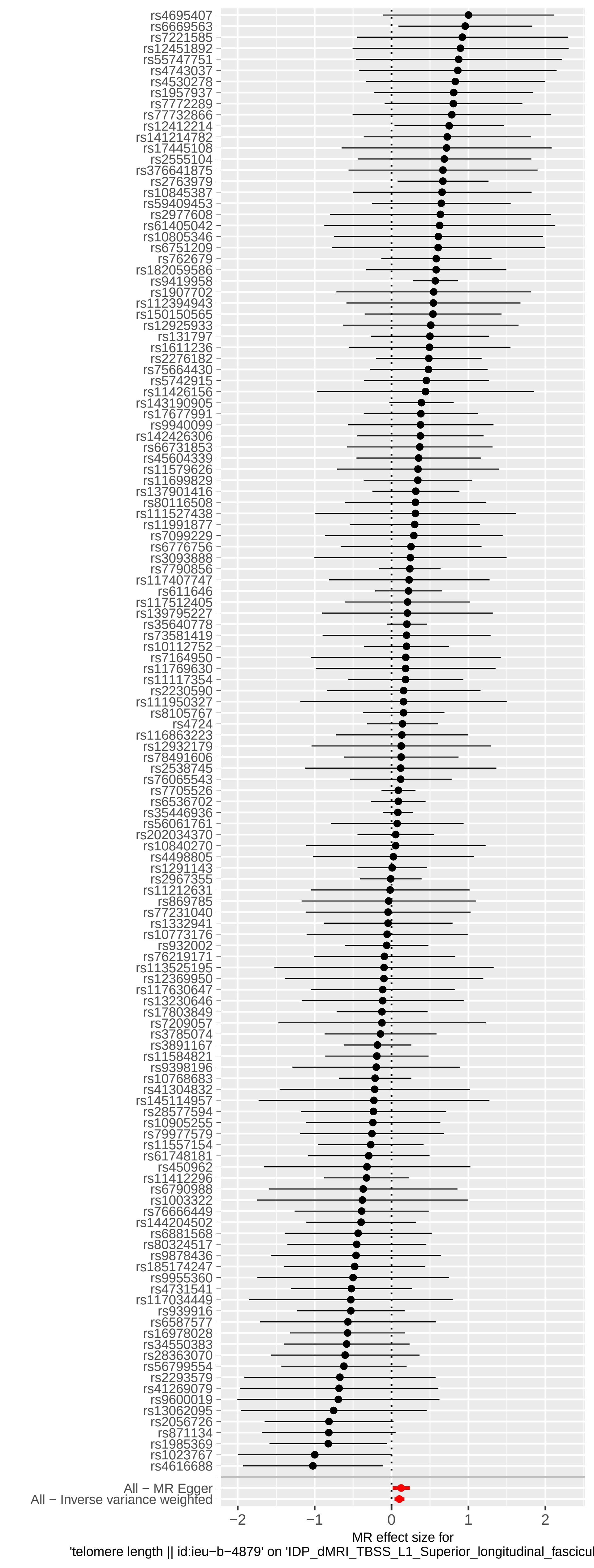
rs4695407
rs6669563
rs7221585
rs12451892
rs55747751
rs4743037
rs4530278
rs1957937
rs7772289
rs77732866
rs12412214
rs141214782
rs17445108
rs2555104
rs376641875
rs2763979
rs10845387
rs59409453
rs2977608
rs61405042
rs10805346
rs6751209
rs762679
rs182059586
rs9419958
rs1907702
rs112394943
rs150150565
rs12925933
rs131797
rs1611236
rs2276182
rs75664430
rs5742915
rs11426156
rs143190905
rs17677991
rs9940099
rs142426306
rs66731853
rs45604339
rs11579626
rs11699829
rs137901416
rs80116508
rs111527438
rs11991877
rs7099229
rs6776756
rs3093888
rs7790856
rs117407747
rs611646
rs117512405
rs139795227
rs35640778
rs73581419
rs10112752
rs7164950
rs11769630
rs11117354
rs2230590
rs111950327
rs8105767
rs4724
rs116863223
rs12932179
rs78491606
rs2538745
rs76065543
rs7705526
rs6536702
rs35446936
rs56061761
rs202034370
rs10840270
rs4498805
rs1291143
rs2967355
rs11212631
rs869785
rs77231040
rs1332941
rs10773176
rs932002
rs76219171
rs113525195
rs12369950
rs117630647
rs13230646
rs17803849
rs7209057
rs3785074
rs3891167
rs11584821
rs9398196
rs10768683
rs41304832
rs145114957
rs28577594
rs10905255
rs79977579
rs11557154
rs61748181
rs450962
rs11412296
rs6790988
rs1003322
rs76666449
rs144204502
rs6881568
rs80324517
rs9878436
rs185174247
rs9955360
rs4731541
rs117034449
rs939916
rs6587577
rs16978028
rs34550383
rs28363070
rs56799554
rs2293579
rs41269079
rs9600019
rs13062095
rs2056726
rs871134
rs1985369
rs1023767
rs4616688
All − MR Egger
All − Inverse variance weighted
−2
−1
0
1
2
MR effect size for
'telomere length || id:ieu−b−4879' on 'IDP_dMRI_TBSS_L1_Superior_longitudinal_fascicul
